# Retrospective analysis of risk factors of slide positivity among febrile patients in the Salween river valley of Shan Special Region II, northern Myanmar

**DOI:** 10.1186/s12889-018-5469-7

**Published:** 2018-04-27

**Authors:** Hui Liu, Jian-Wei Xu, Qi-Zhang Xu, Yi-Rou Zeng

**Affiliations:** 1 Yunnan Institute of Parasitic Diseases, Yunnan Provincial Centre of Malaria Research, Yunnan Provincial Key Laboratory of Vector-borne Diseases Control and Research, Yunnan Provincial Collaborative Innovation Center for Public Health and Disease Prevention and Control, Puer, 665000 China; 2 People’s Hospital of Taikang County, Taikang, 475400 Henan Province China; 3 Mengmao County Hospital, Mengmao, Shan Special Region II Myanmar

**Keywords:** Great Mekong Sub-region, Malaria, Risk factor, Salween River Valley, Slide positivity

## Abstract

**Background:**

In Myanmar, epidemiological conditions have been unclear due to a lack of accurate data. In 2014 and 2016, malaria outbreaks occurred in the Shan Special Region II (SSR2). It was reported that these outbreaks were caused by malaria patients from the Salween River Valley (SRV), but further research is needed to confirm these reports. To examine the risks of malaria infection in the SSR2 section of the SRV, this paper offers a retrospective analysis based on the data we collected in 2009.

**Methods:**

A multivariate logistic model was utilized to analyze risk factors associated with the slide positivity of 2009. Results of the investigation in 2009 were compared with updated data.

**Results:**

The number of slide positivity was 91 (24.7%, 95% confidence interval [CI], 20.3–29.4%) among 369 people who had fever 2 weeks ago of the survey, including 74 (20.1%; 95%CI, 16.1–24.5%) cases of *P. falciparum*, 13 (3.5%; 95%CI, 1.9–5.9%) of *P.vivax* and 4 (1.1%, 95%CI, 0.3–2.8%) of *P. malariae.* The adjusted odds ratio (OR) was 99.8 (95% CI, 24.7–887.7) for patients’ age < 15 years, 6.61 (95%CI, 3.57–10.49) for people living at an altitude of < 800 m, 6.35 (95%CI, 2.45–23.27) for people lacking knowledge on malaria transmission and knowledge on symptoms, 2.10 (95%CI, 1.22–5.11) for people taking no measures against mosquito bites and 5.55 (95%CI, 2.65–13.05) for people delaying treatment. Compared with annual parasitic incidences 13.80 per 10,000 person-years (422/305733) in 2014, 2.36 per 10,000 person-years (73/309004) in 2015 and 5.25 per 10,000 person-years (164/312310) in 2015, malaria burden is reduced.

**Conclusion:**

Age, lower altitude, a lack of knowledge about malaria transmission and symptoms, inaction of measures against mosquito bites and delayed treatment-seeking were independent risk factors for slide positivity. These results indicate that malaria transmission was likely within housing settlements in the SRV, and that the transmission rates within the SRV are higher than in other areas. In order to eliminate malaria, it is important for people to obtain qualified treatment to contain artemisinin resistance.

**Trial registration:**

Trial registration number: ChiCTR-COC-17012522.

Retrospectively registered 31 August 2017.

## Background

Malaria is a disease caused by parasites of the *Plasmodium* family and is transmitted by female *Anopheles* mosquitoes. Remarkable progress has been made in malaria control over the past decade, but malaria is still devastatingly affecting human health and livelihoods globally [1]. About 214 million (range 149–303 million) new cases of malaria were identified in 2015, with the number in the South-East Asia Region (10%) ranking second highest [2]. The Greater Mekong Subregion (GMS) includes Cambodia, Myanmar, Thailand, Vietnam, Laos and Yunnan Province of China. Malaria elimination faces daunting challenges as malaria epidemiology exhibits enormous complexity in the region [3]. Around 70% of the total population in the GMS is still at risk of contracting malaria and the disease is concentrated mainly in remote areas [4]. Although malaria is well controlled in China and Thailand, a high prevalence of malaria is still seen in Myanmar. In response to the recent emergence of artemisinin-resistant malaria in the GMS, Myanmar has become the main focus in the region. However, further micro epidemiological data from northern Myanmar is needed [5].

The World Health Organization (WHO) plans to eliminate malaria by 2030 in all GMS countries [6]. By 2013, we had reduced the malaria burden significantly along the China-Myanmar border [7–9] However, the report of an outbreak of *P. falciparum* malaria marked the first patient infected with malaria in a private rubber plantation importing from the Salween River Valley, Shan Special Region II (SSR2) in June 2014 [10]. Some other malaria outbreaks were reported in 2016 as well (not published). Meanwhile, Department of Health of SSRII registered that the total number of confirmed malaria cases in 2014, 2015 and 2016 were 442, 73, and 164 respectively. From above evidence, we infer that the Salween River Valley may still be one of the current malaria foci. To support this claim, this manuscript presents the results of slide positivity and associated factors before the intensive interventions of the Global Fund to fight AIDS, Tuberculosis and Malaria (GFATM) program. It then discusses implications of the findings for contemporary malaria control and elimination in the GMS.

## Methods

### Study site

**Fig. 1:**
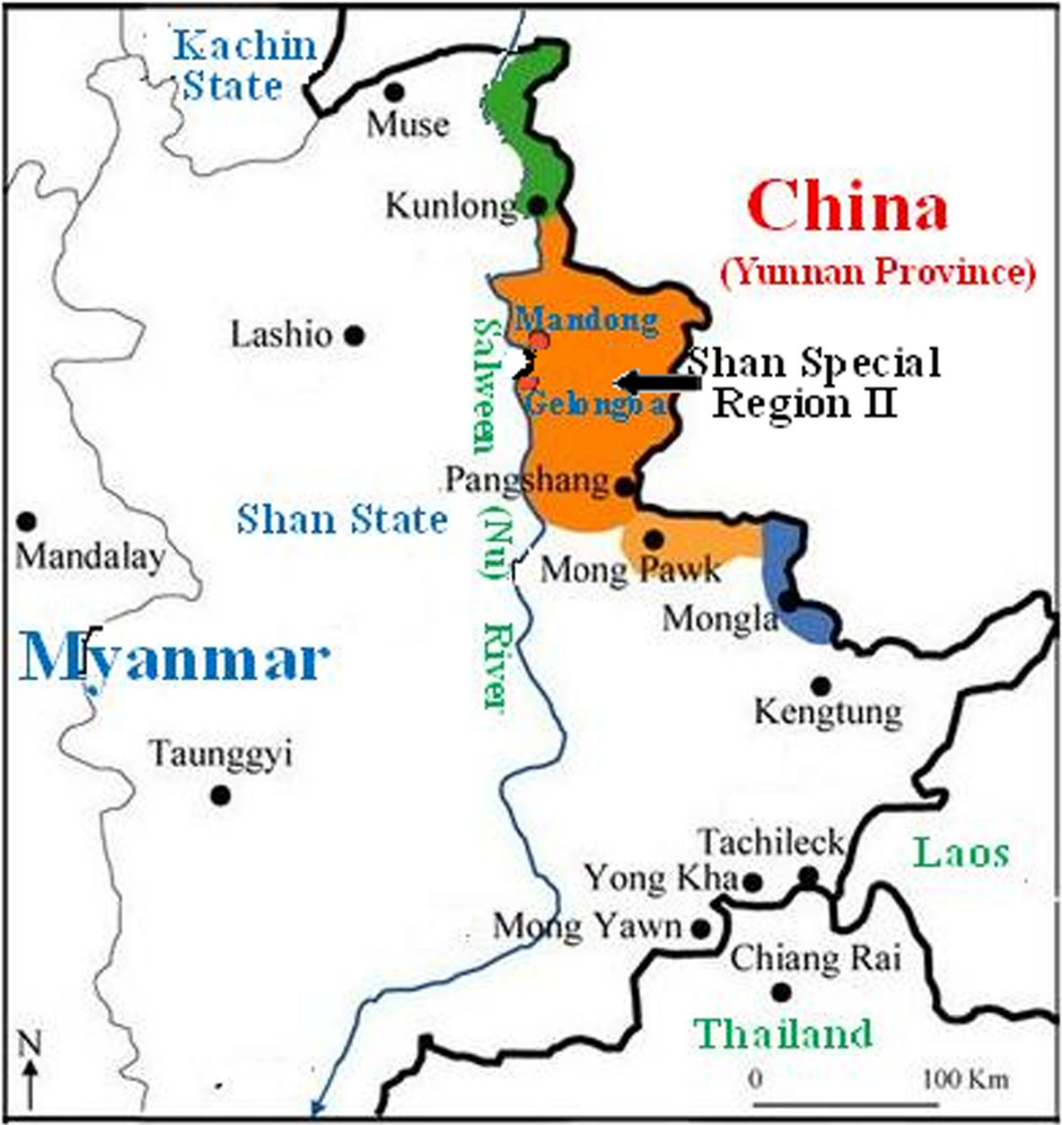
Study site relative to neighboring areas

The SSR2 is known locally as Wa State and the main population are of Wa ethnicity. In the SSR2, malaria (*An. minimus* is the principal vector) is able to be transmited throughout the year with a peak during the later rainy season from September to November [11]. Gelongba and Mandong are two collective settlements in the Salween river valley, SSR2 (Fig. 1). All the health facilities during the investigation are located in Gelongba and Mandong, including a community health centre, two NGO health posts by Aide Medical International (AMI) and two private clinics.

### Sampling and investigation

Based on malaria endemicity and prevalence in the SSR2, we specifically conducted an active detection at the two settlements from October 1st to December 31st, 2009. We sampled 64 villages, with an approximate total population of 18,940. All those who had a self-reported fever in the previous 2 weeks were enrolled in the study after obtaining informed consent [12].

Thick and thin blood smears were prepared for each participant. Two expert microscopists stained malaria blood films with Giemsa and examined by microscopy later. Data on risk factors was collected by interviewing each household head face-to-face with a structured questionnaire (in Chinese). One researcher (Zeng) who could understand both Wa and Chinese Language conducted the interviews in Wa language and filled out the questionnaires in Chinese [12]. Together with the microscopy-diagnosis results, we recorded details of a range of factors contributing to the patient’s risk. This included information concerning age and gender of febrile patients, altitude of their house location, family size, family decisions, annual average cash income, malaria knowledge of household heads, use of any measures against mosquito bites, distance from health facilities and time between onset of illness and seeking treatment.

Each *Plasmodium falciparum* positive individual was treated by administering Dihydroartemisinin-piperaquine tablets (40 mg base dihydroartemisinin and 320 mg piperaquine phosphate per tablet). DAPQ was given once a day for 3 days, based on the recommendation of Ministry of Health, China [13]. *Plasmodium vivax* objects were treated by Chloroquine (CQ) and primaquine (PQ). CQ was administered once a day for 3 days with a total target dose of 24 mg base/kg and PQ was once a day for 8 days with a dose of 0.45 mg base/kg/day [13, 14].

### Data management and analysis

We checked the completeness of the data and then entered and validated them in EpiData (version 3.1). Epitable Calculator of EPI6.04 was used to calculate the slide positivity rates and binomial 95% confidence intervals (CI). A multivariate logistic model was used to calculate odds ratios for risk factors associated with the slide positivity. In the model, we chose the results of microscopy as the outcome (dependent) variable. We then chose sex, age, income, family size, altitude, malaria knowledge, mosquito prevention action, family decision, distance from health facilities and treatment seeking as independent variables [12]. Based on a comprehensive analysis of landform and vegetation, an altitude distribution (< 800 m; 800–1200 m, > 1200 m) was established for the study site: a) areas of < 800 m are usually foothills, most of which are covered by rubber plantations; b) areas 800 to 1200 m are usually mid hills, most of which are crop (such as dry rice and corn) fields; and areas of > 1200 m are usually hills, most of which are forested [12]. Finally, the results of the 2009 investigation were compared with the annual parasitic incidences (API) in recent years.

## Results

### Subject characteristics

We visited a total of 718 households and saw 3678 people. After obtaining informed consent, 369 people who had a self-reported fever in the previous 2 weeks were tested by microscopy. The median age of the participants was 23.7 years (range: 3 months - 69 years) and the male/female sex ratio was 1.6 (Table 1).

**Table 1:** Characteristics and slide positivity among febrile patients in the Salween River Valley of Shan Special Region II, Myanmar

	No. tested (*n* = 369)	No. *P. falciparum* (%, 95%CI)	No. *P. vivax* (%, 95%CI)	No. *P. malariae* (%, 95%CI)	*Plasmodium spp.* (%, 95%CI)
Demographics					
Male	227	37 (16.3, 11.7–21.8)	10 (4.4, 2.1–8.0)	3 (1.3, 0.3–3.8)	50 (22.0, 18.8–28.0)
Female	142	37 (26.1, 19.1–34.1)	3 (2.1, 0.4–6.0)	1 (0.7, 0.0–3.9)	41 (28.9, 21.6–37.1)
Age (years)					
<5	12	10 (83.3, 51.6–97.9)	1 (8.3, 0.2–38.5)	1 (8.3, 0.2–38.5)	12 (100, 73.5–100)
5–15	29	25 (86.2, 68.3–96.1)	2 (6.9, 0.8–22.8)	0 (0, 0–11.9)	27 (93.1, 77.2–99.2)
16–50	276	36 (13.0, 9.3–17.6)	9 (3.3, 1.5–6.1)	3 (1.1, 0.2–3.1)	48 (17.4, 13.1–24.4)
> 50	52	3 (5.8, 1.2–15.9)	1 (1.9, 0.1–11.5)	0 (0, 0–6.8)	4 (7.7, 2.1–18.5)
Annual average cash income (US$)					
≤ 100	191	44 (23.0, 17.3–29.7)	7 (3.7, 1.5–7.4)	2 (1.0, 0.1–3.7)	53 (27.7, 21.5–34.7)
101–200	134	26 (19.4, 13.1–27.1)	5 (3.7, 1.2–8.5)	1 (0.7, 0.02–4.1)	32 (23.9, 16.9–32.0)
> 200	40	4 (10.0, 2.8–23.7)	1 (2.5, 0.1–13.2)	1 (2.5, 0.1–13.2)	6 (15.0, 5.7–29.8)
Family size of the household					
≤ 3	75	12 (16.0, 8.6–26.3)	6 (8.0, 3.0–16.6)	1 (1.3, 0.03–7.2)	19 (25.3, 16.0–36.7)
4–5	108	33 (30.6, 22.1–40.2)	5 (4.6, 1.5–10.5)	2 (1.9, 0.2–6.5)	40 (37.0, 27.9–46.9)
≥ 6	186	29 (15.6, 10.7–21.6)	2 (1.1, 0.1–3.8)	1 (0.5, 0–3.0)	32 (17.2, 12.1–23.4)
Altitude of residence (meters)					
< 800	117	49 (41.9, 32.8–51.4)	4 (3.4, 0.9–8.5)	3 (2.6, 0.5–7.3)	56 (47.9, 38.5–57.3)
800–1200	143	6 (4.2, 1.6–8.9)	7 (4.9, 2.0–9.8)	1 (0.7, 0–3.8)	14 (9.9, 5.5–16.0)
>1200	110	19 (17.3, 10.7–25.7)	2 (1.8, 0.2–6.4)	0 (0, 0–3.3)	21 (19.1, 12.2–27.7)
Malaria knowledge of household heads					
No malaria knowledge of cause and symptoms	52	17 (32.7, 20.3–47.1)	5 (9.6, 3.2–21.0)	0 (0, 0–6.8)	22 (42.3, 28.7–56.8)
Knowing malaria symptoms	240	53 (21.1, 17.0–27.9)	6 (2.5, 1.0–5.4)	3 (1.3, 0.3–3.6)	62 (25.8, 20.4–31.9)
Knowing mosquitoes	77	4 (5.2, 1.4–12.8)	2 (2.6, 0.3–9.2)	1 (1.3, 0.04–7.0)	7 (9.1, 3.7–17.8)
Use of measures against mosquito bite					
No	172	47 (27.3, 20.8–34.6)	8 (4.6, 2.0–9.7)	2 (1.2, 0.1–4.1)	57 (33.1, 26.2–40.7)
Yes	197	27 (13.7, 9.2–19.3)	5 (2.5, 0.8–5.8)	2 (1.0, 0.1–3.6)	34 (17.3, 12.3–23.3)
Family decision					
Husband	283	67 (23.7, 18.8–29.1)	8 (2.8, 1.2–5.5)	2 (0.7, 0.1–2.5)	77 (27.2, 22.1–32.8)
No response	11	2 (18.2, 2.3–51.8)	2 (18.2, 2.3–51.8)	1 (9.1, 0.2–41.3)	5 (45.5, 16.7–76.6)
Wife or co-decision	75	5 (6.7, 2.1–14.9)	3 (4.0, 0.8–11.2)	1 (1.3, 0.03–7.2)	9 (12.0, 5.6–21.6)
Distance from health facilities or drug shops					
> 3 km	295	66 (22.4, 17.7–27.6)	11 (3.7, 1.9–6.6)	4 (1.4, 0.4–3.4)	81 (27.5, 22.4–32.9)
≤ 3 km	74	8 (10.8, 4.8–20.2)	2 (2.7, 0.3–9.4)	0 (0, 0–4.9)	10 (13.5, 6.7–23.5)
Time between onset of illness and seeking treatment (hours)					
Never sought treatment	46	17 (37.0, 23.2–52.5)	4 (8.7, 2.4–20.8)	3 (6.5, 1.4–17.9)	24 (52.2, 36.9–67.1)
> 48	181	48 (26.5, 20.2–33.6)	6 (3.3, 1.2–7.1)	1 (0.6, 0–3.0)	55 (30.4, 23.8–37.6)
25–48	24	2 (8.3, 1.0–27.0)	2 (8.3, 1.0–27.0)	0 (0, 0–14.2)	4 (16.7, 4.7–37.4)
≤ 24	118	7 (5.9, 2.4–11.8)	1 (0.8, 0.02–4.6)	0 (0, 0–3.1)	8 (6.8, 3.0–12.9)
Total	369	74 (20.1, 16.1–24.5)	13 (3.5, 1.9–5.9)	4 (1.1, 0.3–2.8)	91 (24.7, 20.3–29.4)

### SRV blood positivity, 2009 and SSR2 malaria situation, 2014–2016

Out of 369 slides prepared in the investigation in 2009, 91 (24.7%) were positive by microscopy and 74 (81.3% of positive slides), 13 (14.3%) and four (4.4%) of positive slides were *P. falciparum*, *P.vivax* and *P. malariae*, respectively. The ratio of *P. falciparum* versus *P. vivax* was 5.7. We discovered that a high slide positivity rate and a high proportion of *P. falciparum* might be characteristics of low-altitude regions, especially for children under the age of 5 years who were all parasitic positivity and 10 out of 12 children were infected with *P. falciparum*. The API were 13.80 per 10,000 person-years (422/305733) in 2014, 2.36 per 10,000 person-years (73/309004) in 2015 and 5.25 per 10,000 person-years (164/312310) in 2016. Additionally, the ratios of *P. falciparum* versus *P. vivax* in 2014, 2015 and 2016 were 1.13 (224/198), 0.70 (30/43) and 0.38 (45/119), respectively.

### Risk factors for slide positivity

We identified five independent risk factors associated with slide positivity. First, the slide positivity rate for children (< 15 years) was 95.1% (95%CI: 83.5–99.4%). As much as 89.7% (95%CI: 75.8–99.1%) of child positive slides were *P. falciparum*; the adjusted odds ratio (AOR) was 99.8 (95%CI: 24.7–887.7). Second, people living in low-altitude regions (< 800 m) had significantly higher slide positivity than those living in mid-altitude regions (≥800 m) (AOR 6.61[95%CI: 3.57–10.49]). Third, those whose household heads who did not know that malaria was transmitted by mosquitoes and had little awareness of the clinical symptoms of malaria had higher slide positivity (AOR 6.35[95%CI: 2.45–23.27]). Fourth, febrile patients who did not use any measures against mosquito bites had higher incidences of malaria infection (AOR 2.10 [95%CI: 1.22–5.11]). Finally, people who did not seek treatment within 48 h had a higher slide positivity rate (AOR5.55 [95%CI: 2.65–13.05]) (Table 2).

**Table 2:** Risk factors of slide positivity in the Salween River Valley of Shan Special Region II, Myanmar

	Slide positivity (%, 95%CI)	Univariate OR (95% CI)	*P* values	Adjusted OR (95% CI)	*P* values
Sex					
Male (*n* = 227)	50 (22.0, 18.8–28.0)	0.70 (0.42–1.16)	0.1737	0.81 (0.37–1.26)	0.1936
Female (*n* = 142)	41 (28.9, 21.6–37.1)	1		1	
Age (years)					
< 15(*n* = 41)	39 (95.1, 83.5–99.4)	103.5 (25.0–895.8)	< 0.0001	99.8 (24.7–887.7)	< 0.0001
≥ 16(*n* = 328)	52 (15.8, 12.1–20.3)	1		1	
Annual average income (US$)					
≤ 100(*n* = 191)	53 (27.7, 21.5–34.7)	1.37 (0.83–2.29)	0.2371	1.52 (0.81–2.45)	0.2475
≥ 101(*n* = 174)	38 (21.8, 15.9–28.7)	1		1	
Family size of the household					
≤ 5(*n* = 183)	59 (32.2, 25.5–39.5)	2.29 (1.37–3.87)	0.0012	0.89 (0.67–3.93)	0.1819
≥ 6 (*n* = 186)	32 (17.2, 12.1–23.4)	1		1	
Altitude of residence (meters)					
< 800 (*n* = 117)	56 (47.9, 38.5–57.3)	5.69 (3.32–9.79)	< 0.0001	6.61 (3.57–10.49)	< 0.0001
≥ 800 (*n* = 252)	35 (13.9, 9.9–18.8)	1		1	
Malaria knowledge of household heads					
No malaria knowledge of cause and symptoms (*n* = 52)	22 (42.3, 28.7–56.8)	7.33 (2.62–22.22)	0.00002	6.35 (2.45–23.27)	0.0001
Only knowing malaria symptoms (*n* = 240)	62 (25.8, 20.4–31.9)	3.48 (1.49–9.43)	0.0033	2.98 (1.27–10.46)	0.0036
Knowing mosquitoes (*n* = 77)	7 (9.1, 3.7–17.8)	1		1	
Use of measures against mosquito bite					
No (*n* = 172)	57 (33.1, 26.2–40.7)	2.38 (1.42–4.00)	0.0004	2.10 (1.22–5.11)	0.0015
Yes (*n* = 197)	34 (17.3, 12.3–23.3)	1		1	
Family decision					
Husband and no response (*n* = 294)	82 (27.9, 22.8–33.4)	2.83 (1.32–6.76)	0.0069	1.89 (0.68–10.77)	0.0765
Wife or co-decision (*n* = 75)	9 (12.0, 5.6–21.6)	1		1	
Distance from health facilities or drug shops					
> 3 km (*n* = 295)	81 (27.5, 22.4–32.9)	2.42 (1.16–5.54)	0.0152	1.62 (0.66–8.44)	0.0858
≤ 3 km (*n* = 74)	10 (13.5, 6.7–23.5)	1		1	
Time between onset of illness and seeking treatment					
> 48 h and never sought treatment (*n* = 227)	79 (34.8, 28.6–41.4)	5.78 (2.95–12.15)	< 0.0001	5.55 (2.65–13.05)	< 0.0001
≤ 48 (*n* = 142)	12 (8.5, 4.4–14.3)	1		1	

*n* number tested, *95%CI* 95% confidence interval, *OR* odds ratio

## Discussion

Border malaria control is still one of the great challenges in the GMS [3–6]. Reducing malaria endemicity of neighbouring countries largely determines the success of malaria elimination [15–17]. Thus, this paper presents slide positivity and associated risk factors before intensive interventions from China’s GFATM in 2009. The slide positivity rate of febrile patients was 24.7% (91/369) and the ratio of *P. falciparum* versus *P. vivax* was 5.7 (74/13)*.* Additionally, a similar active case detection indicated that the malaria prevalence was even higher in former years. The slide positivity rate was 60% (270/453) and the 270 positive slides includes 245 *P. falciparum*, 18 *P. vivax* and seven *P. malariae* in December 2007 [18]. In comparison of the two active detections, the slide positivity was significantly decreased (x^2^ = 97.7836, *P* < 0.0001) while the ratios of *P. falciparum* versus *P. vivax* were not changed significantly (x^2^ = 4.3554.8, *P* = 1.0369). This shows that a high malaria burden was still present during the investigation. By 2013, the GFATM project decreased the API to 8.60 per 10,000 person-years (260/302496) thereby reducing the malaria burden dramatically in the whole SSR2. This success illustrates the importance of intervention coverage for malaria control [8, 17].

The analysis demonstrates that slide positivity was associated with age (< 15 years), altitude (< 800 m), lack of knowledge on malaria transmission and symptoms, the inaction of measures against mosquito bite and delayed treatment-seeking (> 48 h). A retrospective case-control study documented that the Chinese migrants who stay overnight in the lowland, foothill and half hill areas, especially along the waterside in Myanmar, had higher risks of malaria infection [19]. The difference of slide positivity between male and female is not significant. However, children (< 15 years) had a high slide positivity rate (95.1%) in this study (Table 2). Also, a malaria (i.e. *P. falciparum*) outbreak in 2014 illustrated people aged < 15 years had a high parasite prevalence rate (84.4%) [10]. The demographic characteristics of parasite-positive individuals (children, no difference in sex) suggest that transmission was likely within the housing settlements and also gives evidence of higher than normal transmission in the region. After intervention, if the API is < 1 per 1000 person-year, the program status can transfers from control to elimination, and from there infected cases are mainly imported, male and adult [19, 20]. The findings above highlight the differences of demographic characteristics between endemic and elimination areas.

People’s knowledge, awareness and behaviour may influence their action in self-protection and in seeking treatment, which further affects incidences of malaria infection and willingness to completion of treatment courses [12]. The results highlight that a lack of knowledge on malaria transmission and symptoms, the inaction of measures against mosquito bites and delayed treatment-seeking (> 48 h) were independent risk factors of slide positivity. Decreasing sensitivity of anti-malarial drugs may be attributable to the delayed treatment-seeking and poor compliance to standard treatment course. The genetic diversity of malaria parasites and multiclonal infections are correlated with transmission intensity as well as the development and spread of anti-malarial resistance. High parasite population size and transmission intensity allow effective genetic recombination and mutation of malaria parasites [21, 22]. A sensitivity surveillance of *Plasmodium falciparum* to DAPQ in the two districts from 2007 to 2008 indicates that the fever clearance time (FCT) increased from 23.5 ± 16.97 h in 2007 to 36.0 ± 16.97 h in 2008 (F = 219.4, *P* < 0.0001) and the asexual parasite clearance time (PCT) increased from 23.5 ± 0.71 h in 2007 to 41.0 ± 9.90 h in 2008 (F = 1485.4, P < 0.0001) [23]. The GFATM public health interventions improved local people’s accessibility to qualified malaria prevention and treatment [8]. The FCT and PCT *P. falciparum* malaria cases were 36.4 ± 8.9 h and 53.3 ± 11.3 h in the 2014 outbreak [10]. The FCT has increased 0.4 h between 2008 and 2014 (6 years) but increased 2.5 h between 2007 and 2008 (1 year). The PCT is increased 12.1 h between 2008 and 2014 but increased 17.5 h between 2007 and 2008. The above differences indicate that effective interventions may slow down the declining sensitivity of *P. falciparum* to DAPQ. As resistance of *P. falciparum* to several antimalarial drugs, including ACTs, has reached alarming levels in the GMS, there should be an emphasis on providing qualified treatment, a promotion of treatment-seek behaviour and compliance to cure treatment regimens [8].

Being of a young age might be associated with microscopically confirmed parasitemia because transmission in the children was sufficiently high to provide an increasingly robust immune response in adults. Results are almost certainly underestimated as they bias against those without fever. The true prevalence might be higher than estimated here and the magnitude of such underestimation is due to the prevalence of asymptomatic malaria in this population.

Finally, there remain two lessons from this study that may be of interest to following projects. First, the data used was from an investigation that ran from October 1st to December 31st, 2009. However, the findings obtained after reanalysing the old data and comparing it with the updated data are more meaningful. The analysis confirmed that malaria has not finally been controlled in the SSR2, especially in the Salween River Valley. We still need further investigation and more control of malaria foci in the GMS. Second, the study highly relies on the results of microscopy and a sample of self-report-fever villagers rather than other more reliable technics like molecular methods.

## Conclusion

Independent risk factors for slide positivity are age, altitude, knowledge about malaria transmission and symptoms, self-protection against mosquito bites and treatment-seeking in the SRV of the SSR2. These five factors determined how likely the transmission of malaria was within the housing settlements and also helped researchers predict where higher than the normal transmission was likely to occur in the region. The reduced malaria burden of the SSR2 in recent years shows that promoting people’s access to qualified treatment and promotion of treatment seeking behaviours are important for the malaria elimination and containment of artemisinin resistance.

## Abbreviations

95% CI: Confidence interval
ACT: Artemisinin based therapy
AMI: Aide Medical International
AOR: Adjusted odds ratio
API: Annual parasitic incidences
CQ: Chloroquine
DAPQ: Dihydroartemisinin- piperaquine
FCT: Fever clearance time
GFATM: Global Fund to fight AIDS, Tuberculosis and Malaria
GMS: Greater Mekong Subregion
NGO: Nongovernment organization
OR: Odds ratio
PCT: Parasite clearance time
PQ: Primaquine
SRV: Salween River Valley
SSR2: Shan Special Region II
WHO: World Health Organization
